# Perivascular and Perineural Pathways Involved in Brain Delivery and Distribution of Drugs after Intranasal Administration

**DOI:** 10.3390/pharmaceutics11110598

**Published:** 2019-11-12

**Authors:** Jeffrey J. Lochhead, Thomas P. Davis

**Affiliations:** Department of Pharmacology, University of Arizona, Tucson, AZ 85721, USA; davistp@email.arizona.edu

**Keywords:** intranasal, drug delivery, olfactory nerve, trigeminal nerve, perineural space, perivascular space

## Abstract

One of the most challenging aspects of treating disorders of the central nervous system (CNS) is the efficient delivery of drugs to their targets within the brain. Only a small fraction of drugs is able to cross the blood–brain barrier (BBB) under physiological conditions, and this observation has prompted investigation into the routes of administration that may potentially bypass the BBB and deliver drugs directly to the CNS. One such route is the intranasal (IN) route. Increasing evidence has suggested that intranasally-administered drugs are able to bypass the BBB and access the brain through anatomical pathways connecting the nasal cavity to the CNS. Though the exact mechanisms regulating the delivery of therapeutics following IN administration are not fully understood, current evidence suggests that the perineural and perivascular spaces of the olfactory and trigeminal nerves are involved in brain delivery and cerebral perivascular spaces are involved in widespread brain distribution. Here, we review evidence for these delivery and distribution pathways, and we address questions that should be resolved in order to optimize the IN route of administration as a viable strategy to treat CNS disease states.

## 1. Introduction

The effective delivery of neurotherapeutics is hindered by the low permeability of the vasculature in the central nervous system (CNS). The blood–brain barrier (BBB) is comprised of tight junctions of integral proteins between endothelial cells that form a high transendothelial electrical resistance (TEER) and limit the passage of most substances through the paracellular route [1,2,3,4]. In addition to forming tight junctions, cerebral endothelial cells express a number of key drug transporters at the luminal plasma membrane that restrict the transendothelial passage of many drugs from the blood into the CNS [5,6]. Molecules able to cross the BBB under physiological conditions are typically small (<600 Da) and lipophilic. Indeed, it has been estimated that up to 98% of all small molecules are unable to cross the BBB [7]. These obstacles have led some to propose routes of drug administration that can potentially bypass the blood–CNS barriers and deliver drugs directly to their targets in the CNS.

Intraparenchymal and intrathecal infusions are able to deliver drugs directly into the brain parenchyma or cerebrospinal fluid (CSF), respectively, but these routes of administration are highly invasive, expensive, and not practical for chronic administration. Though the intranasal (IN) route of administration has long been used to deliver drugs to the systemic circulation, increasing evidence has suggested that intranasally-administered drugs may bypass the BBB and rapidly target drugs to the CNS in a simple, non-invasive manner. A number of studies have now been conducted that compare the brain and blood levels of drugs after IN administration and other routes of administration. A higher AUC_brain_/AUC_plasma_ through the IN route vs. other parenteral routes suggests that a portion of the drug is reaching the CNS through pathways that do not involve the systemic circulation and transport across the BBB. For example, peptides can be found in the CSF of humans within 10 min after IN administration with no change in serum levels, suggesting that peptides can access the CNS through pathways that do not require absorption into the bloodstream [8]. In mice, the AUC_brain_/AUC_plasma_ of insulin is nearly 2000-fold higher after IN administration when compared to subcutaneous administration [9]. Even proteins as large as immunoglobulin G (IgG; 150 kDa) have significantly higher AUC_brain_/AUC_plasma_ after IN administration when compared to intra-arterial administration [10]. 

Intranasally-administered therapeutics have effectively treated many animal models of CNS diseases, and clinical trials are currently underway to deliver peptides such as insulin or oxytocin to treat a number of different neurological disorders. In this review, we examine the evidence for direct brain delivery through the IN route, pathways that substances utilize to reach the brain from the nasal passages, and distribution within the CNS after entry.

## 2. Transport across the Nasal Epithelium

There are four distinct types of epithelium in the nasal passages of mammals: squamous, transitional, respiratory, and olfactory [11]. The respiratory epithelium represents nearly 50% of the total nasal cavity surface area in rodents and over 90% in primates, while the olfactory epithelium represents ~50% in rodents and <10% in primates [11]. The lamina propria, located under the basement membrane of the epithelial surface, contains components of the trigeminal and olfactory nerves that provide anatomical connections between the CNS and the nasal passages [12,13]. Pathways connecting these two cranial nerves to the CNS are the likely routes that intranasally-administered drugs utilize to reach the brain [14,15,16,17]. The first step for drug transport to the CNS after IN administration is to cross the surface of the epithelium, where it can then access these two potential pathways in the lamina propria. 

**Figure 1:**
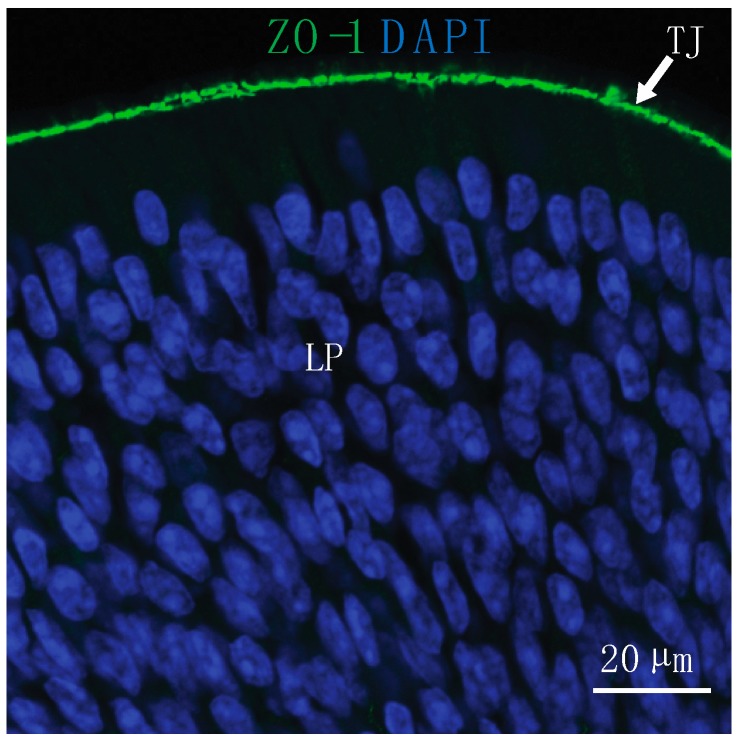
Substances must reach the lamina propria (LP) of the nasal epithelium through paracellular or transcellular routes to access direct pathways to the brain after intranasal (IN) administration. Tight junctions (TJ) located at the epithelial surface present a barrier for IN drug delivery to the central nervous system (CNS). At the olfactory epithelium, TJ are labeled with an antibody to zonula occludens-1 (ZO-1) and nuclei are labeled with 4′,6-diamidino-2-phenylindole (DAPI).

The surface of the olfactory epithelium consists of sustentacular or supporting cells and olfactory sensory neurons (OSN) that send axonal projections to the olfactory bulb through the cribriform plate. Between OSNs and sustentacular cells are tight junction proteins that restrict the paracellular permeability of the epithelium (Figure 1) [18,19]. For example, the olfactory epithelium is less permeable to 10 kDa of dextran than it is to a 3 kDa of dextran [20]. The pre-administration of matrix metalloproteinase-9, which degrades the components of tight junctions and the extracellular matrix, has been shown to increase the permeability of the 10 kDa of dextran and suggests modifying the permeability of the nasal epithelium may allow delivery of larger drugs [20]. 

Molecules that cannot reach the lamina propria through the paracellular route may potentially be transported across the olfactory epithelium by transcytosis. Substances as large as mesenchymal stem cells have been found in the lamina propria after IN administration, but the mechanisms they use to cross the epithelial surface are not well understood [21]. Recently, it has been shown that holes between 5 and 20 μm in diameter can occasionally be observed at the surface of the nasal epithelium [22]. While it has been suggested that these holes may provide a route for IN prion transmission, the spaces at the epithelial surface may also provide access of large therapeutics or stem cells to the lamina propria. The addition of hyaluronidase to the cell suspension may also facilitate passage of cells across the epithelium by degrading hyaluronic acid in the extracellular matrix [21]. 

After transport to the lamina propria from the olfactory epithelium, intranasally-administered drugs may be absorbed into the systemic circulation or into the nasal lymphatic vessels that drain into the cervical lymph nodes [10,16,17,23,24]. The proportion of drugs in the lamina propria that are not absorbed into the blood or cervical lymph nodes may then potentially reach the brain through anatomical pathways (i.e., olfactory and trigeminal) connecting the nasal passages to the CNS. The trigeminal nerve innervates both the respiratory and olfactory regions, suggesting that both regions may be important for IN drug targeting to the brain [14,15,16,17]. There is some evidence, however, suggesting that targeting the olfactory region may more efficiently deliver drugs to the CNS. The brain levels of the small molecules mannitol, nelfinavir, morphine, and fentanyl have been shown to be significantly higher after targeted administration to the olfactory region with a pressurized olfactory device when compared to nose drops deposited primarily on the respiratory epithelium [25,26]. There are also differences in the number and permeability of blood vessels in the respiratory and olfactory regions. Both the number and permeability of blood vessels in the respiratory region are greater than the olfactory region, thus suggesting that drugs targeting the respiratory region are more likely to be absorbed into the systemic circulation than drugs targeting the olfactory region [10]. The absorption of drug into the bloodstream decreases the amount of drug that may reach the brain along the olfactory or trigeminal routes. Additionally, the respiratory epithelium is less permeable to 3 kDa of dextran than the olfactory epithelium [27]. A lower epithelial permeability combined with higher endothelial permeability and vascular density suggests the respiratory epithelium may be a site that is more appropriate to systemically deliver small molecules rather than macromolecules to the CNS.

It should be noted that a variety of substances such as proteins, viruses, and bacteria have been shown to be transported from the nasal passages to the olfactory bulb or brainstem by intracellular uptake and transport within the olfactory nerve or trigeminal nerve, respectively [28,29,30,31,32]. Intracellular transport within axons is a very slow process, however, and is unlikely to describe the pharmacokinetics/pharmacodynamics (PK/PD) observed after intranasal administration [15]. Furthermore, it is unclear how drugs intracellularly transported to the olfactory bulb or brainstem would then distribute to other regions of the brain. Both the rapid delivery and widespread brain distribution of intranasally-administered molecules suggest that extracellular pathways rather than intracellular pathways are more important for the effective brain delivery of drugs. 

## 3. Transport into the Brain

Both the olfactory and trigeminal nerves provide anatomical connections between the CNS and the nasal passages. The axons of OSNs form bundles that travel through foramina in the cribriform plate and synapse on glomeruli in the olfactory bulb [33]. There are also vascular connections between the brain and the nasal passages. The nasal-olfactory artery (a branch of the anterior cerebral artery), for example, sends branches from the olfactory bulb into the olfactory lamina propria [34]. Potential connections between the brainstem and the nasal lamina propria also exist along the trigeminal pathway, as branches V_1_ (ophthalmic) and V_2_ (maxillary) of the trigeminal nerve innervate both the respiratory and olfactory regions of the nasal passages [13,35,36].

Previous studies with radiolabeled tracers have shown the widespread brain distribution of [^125^I]-insulin-like growth factor I (IGF-I) or [^125^I]-interferon-β1b in rats and monkeys, respectively, within 30–60 min following IN administration [16,17]. High levels of each tracer were found in the olfactory bulbs, trigeminal nerves, and brainstem, suggesting that components of the olfactory and trigeminal nerves are involved in the delivery of macromolecules to the brain from the nasal cavity. 

It has previously been shown that substances injected into the brain or CSF drain along cranial nerves in addition to drainage through the arachnoid villi or meningeal lymphatic vessels [33,37,38,39,40,41,42,43,44]. Interestingly, there is some evidence suggesting that pathways involving extracellular fluid movement from the brain along cranial nerves may be bidirectional. For example, when the distribution of potassium ferrocyanide and iron ammonium citrate solutions were compared following either injection into the subarachnoid space or IN administration in rabbits, both routes of administration showed dye located in the perineural spaces of olfactory nerve bundles [45]. Within minutes after IN administration, fluorescent tracers such as dextran (3 kDa), insulin, and IgG can be found associated with olfactory nerve bundles traversing the cribriform plate and in the olfactory nerve layer of the olfactory bulb [10,27,46]. The observation that substances can be found associated with olfactory nerve bundles whether they are administered intranasally or directly into the CNS suggests that extracellular fluid movement across the cribriform plate is bidirectional. Blood vessels that traverse the cribriform plate from the nasal lamina propria also exist [33]. The perivascular spaces (PVS) of these blood vessels are another potential extracellular pathway that drugs may use to enter the CNS, although the mechanisms that regulate fluid dynamics in PVS are controversial and not completely understood [47,48,49].

Immune cells such as monocytes, dendritic cells, and T cells have previously been shown to traffic from the CNS, through the cribriform plate, and to the deep cervical lymph nodes as part of the afferent limb of the CNS immune response [50,51,52]. Recently, it was shown that mesenchymal stem cells can be found crossing the cribriform plate adjacent to the olfactory nerve bundles and in the olfactory nerve layer of the olfactory bulb two hours after IN administration [21]. These observations suggest that cells may be able to both leave and enter the CNS across the cribriform plate. 

Substances injected into the CNS or intranasally-administered can be found in the deep cervical lymph nodes [10,17,37,38,40,42,43]. Lymphatic vessels can also be found traversing the cribriform plate [33,53]. Currently, evidence linking lymphatic vessels to the transport of substances to the CNS after IN administration is lacking. Whether lymphatic vessels near the cribriform plate are involved in the IN delivery of drugs to the CNS is an area that warrants further investigation.

**Figure 2:**
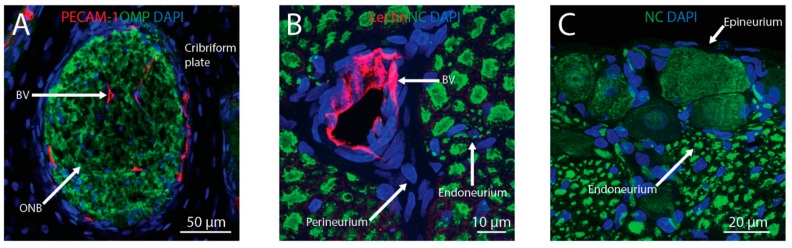
Intranasally-administered therapeutics may reach the brain from the nasal lamina propria through the olfactory or trigeminal pathways. Perineural spaces associated with olfactory nerve bundles (ONB) and perivascular spaces associated with blood vessels (BV) traversing the cribriform plate (**A**) provide anatomical connections between the olfactory bulb and lamina propria. Perivascular spaces and perineural spaces (i.e., in the endoneurium, perineurium, or epineurium) of the trigeminal nerve (**B**,**C**) provide anatomical connections between the brainstem and lamina propria. Blood vessels were labeled with anti-platelet endothelial cell adhesion molecule-1 (PECAM-1) or tomato lectin, ONB were labeled with anti-olfactory marker protein (OMP), axons in the trigeminal nerve were labeled with the Neuro-Chrom (NC) antibody, and nuclei were labeled with DAPI.

In addition to the olfactory pathway, there is evidence that intranasally-administered drugs can reach the CNS along the trigeminal pathway. Fluorescently-labeled insulin or IgG can be detected in the perivascular and perineural spaces of the trigeminal nerve within 30 min after IN administration [10,54]. These potential therapeutics can be found associated with the epineurium, perineurium, and, to a lesser extent, the endoneurium of the trigeminal nerve as well as in PVS. Transport along these extracellular components of the trigeminal nerve as it enters the pons from the nasal passages is likely involved in the brain delivery of drugs to the CNS. Tracers injected into the CSF can also be found associated with the trigeminal nerve, suggesting that the trigeminal pathway may also be involved in both the brain entry and exit of substances [43]. The bulk flow rate within cranial nerves has not been well investigated, but the appearance of tracers in the brainstem within 20 min after IN administration in rats suggests that the bulk flow rate along the trigeminal nerve must be at least 1.0 mm/min [15]. The elucidation of extracellular fluid flow rates within cranial nerves and PVS will be needed to more completely understand the mechanisms regulating IN drug delivery to the CNS. Taken together, the data currently suggest that perineural and/or PVS associated with the cribriform plate and the trigeminal nerves are the pathways that intranasally-administered macromolecules utilize to enter the CNS from the nasal lamina propria (Figure 2).

## 4. Distribution within Brain

Intranasally-administered drugs and tracer molecules typically exhibit widespread brain distribution within minutes. When estimating whether intracellular transport, diffusion, or convective (bulk) flow is the mechanism likeliest to explain the brain pharmacokinetics after IN administration, the data best fit convective flow as the predominant mechanism [15]. In the CNS, convective flow has been shown to occur within the CSF and the extracellular fluid of PVS [49]. Evidence suggests arterial pulsations caused by the cardiac cycle provide the driving force for fluid movement within PVS [55,56,57]. The delivery of intranasally-administered drugs to either the CSF or PVS may allow for widespread brain distribution similar to what has been observed after administration into the cisterna magna.

Though several groups have reported tracer distribution along cerebral PVS after central injections, controversy exists as to whether perivascular fluid flows in the same direction as blood flow, in the opposite direction, or bidirectionally [47,56,57,58,59,60,61]. Ichimura et al. observed that the movement of rhodamine-labeled albumin after injection into a cerebral PVS was “slow and variable” in direction, although these studies were done with an open cranial window that may have influenced intracerebral pressure gradients [58]. It has been suggested that discrepancies in the direction of perivascular fluid flow between groups may be due to tracer moving down its concentration gradient, and this direction appears to be with blood flow when injected into the CSF and against blood flow when injected into the parenchyma [48,62,63,64]. In this scenario, arterial pulsations lead to convective stirring or mixing within PVS rather than unidirectional flow, either with or against the direction of blood flow [49]. Another scenario that has been suggested is that fluid flows with the direction of blood flow in cerebral arteries and against the direction of blood flow in cerebral veins [49,59]. Further complicating the interpretation of the experimental data is the observation that both the presence and type of anesthesia as well as whether the animal is awake or alive during observation can alter the kinetics and location of tracer within cerebral PVS after CSF injection [43,65]. While fluid dynamics within PVS are not fully understood, we feel it is likely bidirectional in nature due to convective mixing, and this possibility suggests that the IN delivery of drugs into the cerebral PVS may allow for distribution throughout the brain. 

**Figure 3:**
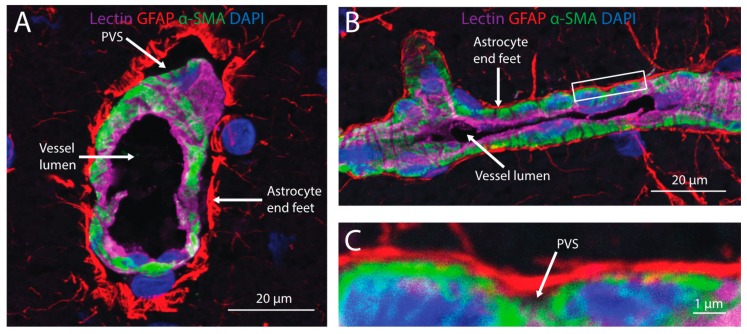
Cerebral perivascular spaces (PVS) provide potential pathways for brain distribution of intranasally-administered therapeutics. A cross-section (**A**) and side view (**B**) of a cortical blood vessel with endothelial cells labeled with lectin (magenta), astrocyte end feet labeled with an anti-glial fibrillary acidic protein (GFAP; red), mural cells labeled with anti-alpha smooth muscle actin (α-SMA) and nuclei labeled with DAPI (blue). A high magnification view (**C**) of the inset in (**B**) shows the PVS located between astrocyte end feet and mural cells ensheathing the blood vessel.

There is some evidence that intranasally-administered tracers are able to access and distribute within the cerebral PVS (Figure 3). High levels of radioactive tracers associated with the arteries of the Circle of Willis have been measured after IN administration and perfusion-fixation in rats and monkeys [10,16,17]. Fluorescently-labeled dextrans have also been observed in PVS of cerebral arteries on the pial surface of the brain as well as vessels within the brain parenchyma shortly after IN administration [20]. These observations suggest that intranasally-administered tracers may distribute along cerebral PVS in a similar manner to tracers administered into the brain parenchyma or CSF. Estimates of bulk flow velocity within cerebral PVS vary widely from 0.01 to 1.1 mm/min [49,57,58,66]. The movement of drugs from PVS to targets in the surrounding brain parenchyma is likely size-dependent (i.e., larger molecules are less able to access the parenchyma from PVS) [59,60]. The possibility that larger drugs may not be able to access targets within the parenchyma from PVS should be taken into consideration when utilizing PVS to deliver drugs through either the IN or intrathecal routes.

Interestingly, entry into the CSF does not seem to precede the brain delivery of intranasally-administered drugs in all instances. For example, [^125^I]-IGF-I, [^125^I]-vascular endothelial growth factor, and [^125^I]-transforming growth factor β1 have all been detected in widespread brain regions but not CSF following IN administration [16,17,24,44]. In these instances, it is possible that the intranasally-administered compound entered the PVS of arteries in the nasal lamina propria that are also continuous with arteries of the olfactory bulb and/or trigeminal nerves. Transport within the PVS of these arteries that branch off other cerebral arteries could then provide a transport network along major arteries and their branches throughout the brain. Though the mechanisms involved in the rapid, widespread brain distribution of intranasally-administered tracers are not fully understood, the evidence to date has suggested that cerebral PVS are prominently involved.

## 5. Conclusions

**Figure 4:**
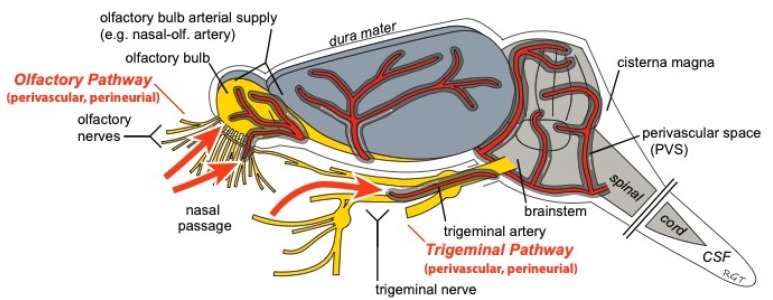
Intranasally-administered drugs may reach the CNS from the nasal passages along perivascular and/or perineural spaces of the olfactory and trigeminal nerves. Once in the CNS, widespread brain distribution may occur along cerebral perivascular spaces (PVS). Figure modified from Lochhead et al. [20].

The IN route of administration has the potential to non-invasively target drugs to the central compartment through direct pathways connecting the nasal passages to the CNS (Figure 4). These characteristics suggest that the IN route may allow patients to easily self-administer potent, non-BBB permeable drugs (e.g., biologics) to the brain while minimizing unwanted systemic side effects. Though therapeutics ranging in size from small molecules to stem cells have successfully been delivered intranasally to treat CNS diseases in pre-clinical models, there are still a number of unresolved questions regarding the pathways and mechanisms regulating IN delivery to the brain.

A better understanding of the permeability characteristics of the olfactory epithelium and the mechanisms involved in transporting substances from the epithelial surface to the lamina propria will allow one to better select/design therapeutics for brain targeting through the IN route. For example, large substances may exhibit a low permeability across the epithelium, while small molecules may easily cross the epithelium but also be more readily absorbed into the systemic circulation. Further research is needed to characterize how perivascular and perineural fluids flow across the cribriform plate, within the trigeminal nerve, and along cerebral PVS. If extracellular fluids in these locations indeed flow in a bidirectional manner, this observation would have important physiological and pharmacological implications. Understanding the anatomical, physiological, and physicochemical limitations of IN drug delivery may ultimately lead to significant advances in drug delivery and the treatment of CNS disorders. The promise of delivering improved and effective therapies to treat the significant number of CNS disease states may well be centered on a future that involves intranasal drug delivery.
